# Noninvasive assessment of liver fibrosis with the aspartate transaminase to platelet ratio index (APRI): Usefulness in patients with chronic liver disease: APRI in chronic liver disease

**Published:** 2011-02-01

**Authors:** Yusuf Yilmaz, Oya Yonal, Ramazan Kurt, Muharrem Bayrak, Bilge Aktas, Osman Ozdogan

**Affiliations:** 1 Department of Gastroenterology, Marmara University, School of Medicine, Istanbul, Turkey; 2 Department of Internal Medicine, Uludag University Medical School, Bursa, Turkey; 3 Department of Internal Medicine, Marmara University, School of Medicine, Istanbul, Turkey

**Keywords:** Chronic hepatitis C, Chronic hepatitis B, Fatty liver, Fibrosis, Aspartate aminotransferases

## Abstract

**Background:**

The aspartate aminotransferases (AST) to platelet ratio index (APRI) may serve as a noninvasive marker to assess liver fibrosis.

**Objectives:**

To assess the diagnostic ability of the APRI for prediction of fibrosis in patients with chronic hepatitis B (CHB), chronic hepatitis C (CHC), and non-alcoholic fatty liver disease (NAFLD).

**Patients and Methods:**

This retrospective study included 207 patients with CHB, 108 with CHC, and 140 patients with NAFLD. The APRI was calculated as (AST level/upper normal limit for AST)/platelet counts (109/L) × 100. The stage of liver fibrosis in patients with chronic viral hepatitis was graded using the METAVIR scale. The Kleiner system for grading fibrosis was used in patients with NAFLD.

**Results:**

Bivariate correlation analyses showed that the APRI was significantly associated with fibrosis scores in patients with CHC (p = 0.2634, p = 0.0059) and NAFLD (p = 0.2273, p = 0.0069), but not in those with CHB (p = 0.1005, p = 0.1495). Receiver operating characteristic (ROC) curves were used for assessing the ability of the APRI as a predictor of the absence or presence of liver fibrosis (fibrosis score of 0 vs fibrosis scores of 1-4). In patients with CHC, the APRI showed a sensitivity of 72.7% and a specificity of 62.4% for detection of fibrosis (p<0.01). In the NAFLD group, the APRI showed a sensitivity of 60.0% and specificity of 73.3% for detection of fibrosis (p<0.01). In patients with CHB, the APRI showed a sensitivity of 55.0% and a specificity of 75.4% for fibrosis (p=NS).

**Conclusions:**

The APRI shows an acceptable accuracy for the assessment of liver fibrosis in patients with CHC and NAFLD, but not in those with CHB.

## Background

Progressive liver fibrosis is the main cause of organ failure in chronic liver diseases of any etiology. Advanced liver fibrosis results in cirrhosis that can in turn lead to liver failure, portal hypertension and hepatocellular carcinoma [1][2]. Fibrosis develops with different spatial patterns and is a consequence of various prevalent mechanisms according to the diverse causes of parenchymal damage. Early detection of fibrosis would allow for initiation of anti-fibrotic therapies capable of halting and even reversing this process. This would in turn prevent progression to hepatic cirrhosis, and the morbidity and mortality this condition entails [3]. To date, liver biopsy remains the gold standard for the evaluation of liver fibrosis. However, its invasiveness, the observations of significant side effect profile, and susceptibility of this technique to sampling error ultimately make it a suboptimal technique [4][5]. For these reasons, it is necessary to find out new, reliable, and non-invasive diagnostic methods for identifying patients with liver fibrosis. Simple biochemical markers to identify hepatic fibrosis are appealing, because they are non-invasive, and repeated testing at regular intervals is more feasible due to lower cost [6]. Several studies have suggested that the aspartate aminotransferases (AST) to platelet ratio index (APRI) may be a useful noninvasive marker of hepatic fibrosis in patients with chronic liver disease [7][8][9][10][11][12]. In addition, preliminary results indicate the potential usefulness of the APRI for predicting significant fibrosis in patients with nonalcoholic fatty liver disease (NAFLD) [9]. Importantly, simple fibrosis scores such as the APRI are derived from routine tests and are suitable for serial assessment of patients during therapy and interventions.

## Objectives

In this retrospective study, we aimed to validate in a Turkish tertiary health care setting the diagnostic usefulness of the APRI in detection of fibrosis in those with chronic hepatitis C (CHC), NAFLD, and chronic hepatitis B (CHB).

## Patients and Methods

All patients were recruited at the Department of Gastroenterology, Marmara University School of Medicine, Istanbul, Turkey. Written informed consent for liver biopsy had been obtained from all the patients. The study protocol was approved by the local Institutional Review Board. The study included 207 patients with CHB, 108 with CHC, and 140 with NAFLD. The diagnosis of CHB or CHC infection was based on typical biochemical data and the detection of anti-HCV antibodies or HBV markers. All patients with CHB were HBsAg-positive, anti-HBs-negative, and HBV DNA-positive. The diagnosis of NAFLD was based on the following criteria: a) ultrasound detection of steatosis more than or equal to grade 1; b) absent-to-low alcohol consumption, i.e. < 30 g/day (men) and < 20 g/day (women); c) exclusion of viral hepatitis B and C, Wilson's disease, α1-antitrypsin deficiency, autoimmune hepatitis, genetic hemochromatosis, and use of steatogenic drugs. The entire study population was negative for other forms of viral hepatitis and human immunodeficiency virus (HIV) infection. Other conditions known to cause liver dysfunction were excluded on the basis of clinical evaluation. All participants underwent liver biopsy. Ultrasonography-guided liver biopsies were performed under conscious sedation using a 16G Tru-cut needle (Quick-Core, Cook-Medical, Bloomington, IN, USA). Before performing the biopsy, we obtained written informed consent from all patients. The specimens were fixed with formalin and embedded in paraffin blocks. Serial sections (sectioned at 4-mm intervals) were stained with hematoxylin-eosin and Masson's trichrome. Liver biopsy specimens were reviewed by one pathologist who was unaware of the patient details and clinical data. Serum (AST) levels were determined using an autoanalyzer (Roche Cobas Integra 800, Roche Diagnostics GmbH, Switzerland). In this study, the normal value of AST ranged from 5 to 40 U/L. The platelet count was performed on an automated hematology analyzer (Sysmex 2100, Roche diagnostic GmbH, Sysmex, Switzerland). The APRI was calculated as [7]:

APRI = (AST level/Upper normal limit for AST / Platelet count (10 /L)) × 100

In patients with chronic viral hepatitis, fibrosis was graded according to the METAVIR scale [13], which grades fibrosis on a five-point scale as follows: F0, no fibrosis; F1, portal fibrosis without septa; F2, portal fibrosis with a few septa; F3, numerous septa without cirrhosis, and F4, cirrhosis. The METAVIR scale has excellent inter-observer reliability. The Kleiner system was used for grading fibrosis in patients with NAFLD [14]. According to the Kleiner system, fibrosis is staged as follows: Stage 0, no fibrosis; stage 1, perisinusoidal or periportal fibrosis; stage 2, perisinusoidal and portal/periportal fibrosis; stage 3, bridging fibrosis; and stage 4, cirrhosis. Bivariate Spearman's rank correlation coefficients (p) were used to assess correlations between the study variables. Receiver operating characteristic (ROC) curves were used to assess the usefulness of the APRI as a predictor of the absence or presence of liver fibrosis (fibrosis score of 0 vs fibrosis scores of 1-4) in each group of patients. All statistical analyses were performed using the MedCalc software package version 7.2 for Windows® (MedCalc, Mariakerke, Belgium). A p-value < 0.05 (2-tailed) was considered statistically significant.

## Results

The general characteristics of the study participants are shown in Table 1. The study included 207 patients with CHB (70 women and 137 men with mean ± SD age of 43.4 ± 12.2 years), 108 patients with CHC (81 women and 27 men with mean±SD age of 53.3 ± 11.5 years), and 140 patients with NAFLD (63 women and 77 men with mean ± SD age of 48.1 ± 8.7 years).

**Table 1:** General characteristics of the study participants

**Characteristic**	**Chronic hepatitis B** (n=207)	**Chronic hepatitis C **(n=108)	**Nonalcoholic fatty liver disease **(n=140)
**Sex **(females/males)	70/137	81/27	63/77
**Age **(years)	43.4 ± 12.2	53.3 ± 11.5	48.1 ± 8.7
**BMI **(kg/mm)	26.9 ± 3.8	27.4 ± 3.9	30.3 ± 4.3
**AST **(U/L) median (interquartile range) a	37 (25)	36 (28)	40 (22)
**GGT **(U/L) median (interquartile range) a	42 (42)	37 (39)	46 (47)
**Platelet **(No/mm3)	224714 ± 62542	221851 ± 75026	249871 ± 59752
**Total cholesterol **(mg/dL)	183 ± 44	177 ± 38	218 ± 41
**HDL cholesterol **(mg/dL)	48 ± 11	49 ± 15	46 ± 9
**Triglycerides **(mg/dL) median (interquartile range) a	101 (84)	94 (68)	166 (123)
**Fibrosis scoremedian **(interquartile range)^a^	1 (2)	1 (2)	1 (2)
**Fibrosis score **(mean ± SEM)	1.14 ± 0.09	1.56 ± 0.12	0.89 ± 0.09
**APRI median **(interquartile range) a	0.46 (0.38)	0.49 (0.43)	0.43 (0.33)

^a^ Interquartile ranges were calculated as the difference between the 75th percentile and the 25th percentile.

**Figure 1:**
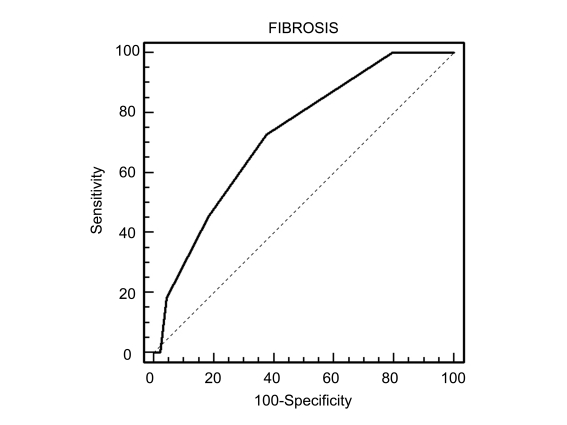
Receiver operating characteristic (ROC) curve of APRI   for prediction of fibrosis according to the Metavir system in ptients   with chronic hepatitis C

**Figure 2:**
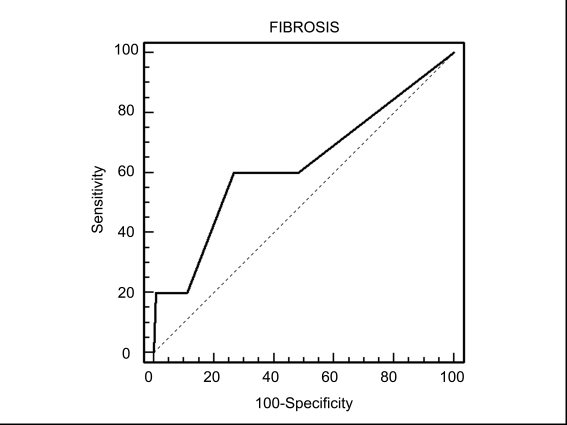
Receiver operating characteristic (ROC) curve of APRI   for prediction of fibrosis according to the Kleiner system in patients with NAFLD

**Figure 3:**
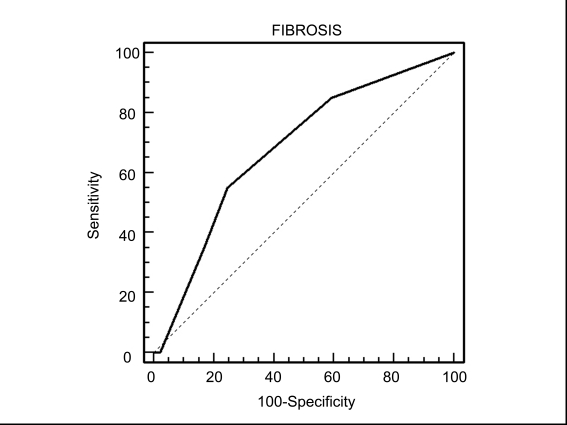
Receiver operating characteristic (ROC) curve of APRI   for prediction of fibrosis according to the Metavir system in patients   with chronic hepatitis B

Bivariate Spearman's rank correlation coefficients were used to assess the association between the APRI and hepatic fibrosis scores. The results showed that the APRI was significantly associated with fibrosis scores in patients with CHC (p = 0.2634, p = 0.0059) and NAFLD (p = 0.2273,p = 0.0069), but not in those with CHB (p = 0.1005, p = 0.1495). ROC curves were used to illustrate the usefulness of the APRI as a predictor of the absence or presence of liver fibrosis (fibrosis score of 0 vs fibrosis scores of 1-4) in each group of patients. In patients with CHC, the APRI (optimal cut-off point > 0.44) showed a sensitivity of 72.7% and a specificity of 62.4% for diagnosis of fibrosis-a fibrosis score of 1-4 (area under the ROC curve = 0.582, standard error = 0.069, 95% CI, 0.519-0.697, p < 0.01, Figure 1). In the NAFLD group, the APRI (optimal cut-off point > 0.45) showed a sensitivity of 60.0% and a specificity of 73.3% for a fibrosis score of 1-4 (area under the ROC curve = 0.627, standard error = 0.063, 95% CI, 0.526-0.721, p < 0.01, Figure 2). In patients with CHB, the APRI (optimal cut-off point > 0.36) showed a sensitivity of 55.0% and a specificity of 75.4% for a fibrosis score of 1-4 (area under the ROC curve = 0.541, standard error = 0.047, 95% CI, 0.457-0.622, p = NS, Figure 3).

## Discussion

Our retrospective study showed that APRI has an acceptable accuracy for the assessment of liver fibrosis in patients with CHC and NAFLD, but not in those with CHB. Our results clearly indicate that the sensitivity of the APRI for the detection of liver fibrosis depends on the etiology of chronic liver disease. Hepatic fibrosis, a reparative response to various types of liver injury, has emerged as the primary determinant of the outcome in advancing chronic liver disease, including cirrhosis [3][4][5][6]. In light of the dramatic increase in the prevalence of NAFLD, noninvasive, simple, reproducible, and reliable biomarkers that can allow identifying patients with liver fibrosis are greatly needed [6]. The availability of such biomarkers has tremendous potential to radically alter the diagnostic and monitoring strategies through the reduction in the need for liver biopsy [4][15] The APRI is based on routine laboratory test results and is therefore readily available in the clinical practice. This index has been reported to predict the presence of significant fibrosis and cirrhosis in several investigations [7][8][9][10][10][12]. However, most of the studies were conducted in Western Europe or in the US. Therefore, it is questionable whether the APRI may be used in the noninvasive assessment of fibrosis within a broad spectrum of chronic liver diseases in Turkey. Wai, et al, [7] derived and validated the APRI in a cohort of 270 patients with CHC. In their study, the area under the ROC curves of the APRI for predicting significant fibrosis and cirrhosis were 0.80-0.88 and 0.89-0.94, respectively. Based on the high predictive values, the authors concluded that the APRI can obviate liver biopsy in approximately 50% of patients [7]. Subsequently, numerous researchers have attempted to validate these findings but the results were conflicting [16][17][18]. The differences in patient populations, including the prevalence of significant fibrosis, and in the reference ranges for AST may in part explain these discrepancies. In a recent systematic review [12], it has been shown that the areas under the ROC curves of the APRI for predicting HCV-related significant fibrosis and cirrhosis were 0.76 (95% CI: 0.74-0.79) and 0.82 (95% CI: 0.79-0.86), respectively. In keeping with previous data, the results of our study showed that APRI had a sensitivity of 72.7% and a specificity 62.4% for predicting fibrosis in patients with chronic HCV infection. Loaeza-del-Castillo, et al, have recently shown that the APRI tend to increase with the degree of fibrosis in patients with NAFLD [9]. Calès and coworkers also observed that the use of APRI produced an area under the ROC curve of 0.943 in patients with NALFD [19]. These results are in good agreement with our data and suggest that APRI may be a useful noninvasive marker of liver fibrosis in this group of patient. The prevalence of NAFLD appears to be increasing worldwide, in part due to the increasing numbers of adult and pediatric individuals who have the metabolic syndrome [20][21]. Although only a minority of patients with NAFLD progress to clinically important stages of fibrosis, early identification is paramount to prevent cirrhosis and liver cancer [20]. In the present retrospective study, we did not find a statistically significant association between the APRI and fibrosis in patients with CHB. In addition, the sensitivity of APRI for the detection of fibrosis in this group of patients was not statistically significant according to ROC curve analysis. Our results are in agreement with those of Elloumi, et al, [22] and Sebastiani, et al, [23] who provided evidence that APRI was not useful in predicting histology in chronic hepatitis B patients. Similarly, Al-Mahtab, et al, [24] reported that the APRI does not appear to be of use in predicting fibrosis in patients with CHB. It is likely that the lack of correlation between the APRI and fibrosis in CHB is due to the platelet count. CHC [25] and NAFLD [26] have been associated with low platelet counts, and this might explain the usefulness of this index in these clinical entities. In contrast, the APRI does not clearly show a diagnostic value for fibrosis in CHB that would be considered adequate by many clinicians. However, some conflicting data exist [27]. Different number and characteristics of subjects recruited and ethnical differences (Turkish subjects in this report vs Koreans' [27]) might at least partially explain the differences among the studies. As the HBV seroprevalence rate is known to be 25%-60% in Turkey with the highest prevalence in the East and South-east [28], it would be important to devise noninvasive tools for assessing the degree of liver fibrosis in this clinical entity. In this regard, future studies should investigate the potential usefulness of other noninvasive tests such as FIB-4 and FORNS biochemistry indices as well as transient elastography [29][30][31]. In conclusion, the results of our study confirm and expand previous findings showing that the APRI has an acceptable accuracy for the assessment of liver fibrosis in patients with CHC and NAFLD but not in those with CHB. Further studies involving a greater number of patients are warranted to validate the usefulness of APRI compared with other noninvasive marker of fibrosis in clinical practice.
